# Effect of cyclic deformation on xenogeneic heart valve biomaterials

**DOI:** 10.1371/journal.pone.0214656

**Published:** 2019-06-13

**Authors:** Ailsa J. Dalgliesh, Mojtaba Parvizi, Christopher Noble, Leigh G. Griffiths

**Affiliations:** 1 Department of Veterinary Medicine: Medicine and Epidemiology, University of California, Davis, Davis, CA, United States of America; 2 Department of Cardiovascular Diseases, Mayo Clinic, SW, Rochester, MN, United States of America; Universidad de Navarra, SPAIN

## Abstract

Glutaraldehyde-fixed bovine pericardium is currently the most popular biomaterial utilized in the creation of bioprosthetic heart valves. However, recent studies indicate that glutaraldehyde fixation results in calcification and structural valve deterioration, limiting the longevity of bioprosthetic heart valves. Additionally, glutaraldehyde fixation renders the tissue incompatible with constructive recipient cellular repopulation, remodeling and growth. Use of unfixed xenogeneic biomaterials devoid of antigenic burden has potential to overcome the limitations of current glutaraldehyde-fixed biomaterials. Heart valves undergo billion cycles of opening and closing throughout the patient’s lifetime. Therefore, understanding the response of unfixed tissues to cyclic loading is crucial to these in a heart valve leaflet configuration. In this manuscript we quantify the effect of cyclic deformation on cycle dependent strain, structural, compositional and mechanical properties of fixed and unfixed tissues. Glutaraldehyde-fixed bovine pericardium underwent marked cyclic dependent strain, resulting from significant changes in structure, composition and mechanical function of the material. Conversely, unfixed bovine pericardium underwent minimal strain and maintained its structure, composition and mechanical integrity. This manuscript demonstrates that unfixed bovine pericardium can withstand cyclic deformations equivalent to 6 months of *in vivo* heart valve leaflet performance.

## Introduction

Valvular heart disease (VHD) accounts for substantial morbidity and mortality in developed countries such as the United States, where the population prevalence of moderate to severe VHD is approximately 2.5% [1]. Furthermore, VHD incidence increases with age reaching approximately 13.2% in patients 75 years and older. Consequently, VHD currently affects 5 million Americans and results in 600,000 fatalities per annum [2]. Currently, surgical valve replacement is the only clinically proven long-term treatment for VHD, with 100,000 valve replacements performed annually in the United States [1]. Although both mechanical and biological heart valves are available, biological heart valves (BHVs) offer superior hemodynamics and eradicate the requirement of lifelong anticoagulation therapy [1, 3]. Unfortunately, current BHVs are plagued with chronic immune response and fatigue-induced structural deterioration that limit valve life expectancy to approximately 10 years in adults and less in pediatric patients [4, 5]. The deficiencies of current bioprosthetic heart valve leaflet materials led the National Heart, Lung, and Blood Institute to recognize the necessity for development of improved heart valve replacement biomaterials that can overcome these debilitating limitations [6].

The average heart valve undergoes approximately 3 billion cycles of opening and closing throughout the patient’s lifetime. As a result, heart valve leaflets are exposed to hemodynamic pressure, cyclic flexure and fluid flow, resulting in cyclical shear, compression and tensile stress on the material [7]. To determine if biological heart valve (BHV) replacement leaflets are capable of withstanding the stresses placed on native valves *in vivo*, Food and Drug Administration (FDA) currently dictates 200 million deformation cycles under physiologic conditions in a heart valve accelerated wear tester, representing approximately 6 years of *in vivo* function [7]. However, such testing assumes that the biomaterial is inert *in vivo*, undergoing neither destructive nor constructive remodeling due to recipient cellular repopulation. This assumption is largely true for current BHVs, since the glutaraldehyde-fixation process renders the biomaterial incompatible with acute recipient cellular repopulation, repair and remodeling [8]. However, chronic graft-specific destructive immune response towards glutaraldehyde-fixed tissues persists, resulting in cumulative damage throughout the BHV’s lifetime [5]. Furthermore, inability to undergo constructive recipient cellular repopulation and remodeling renders current BHV leaflets unable to repair chronic cumulative damage caused by *in vivo* biological (e.g., chronic immune responses) and mechanical (e.g., cyclic loading) insults. Consequently, due to the requirement for glutaraldehyde fixation, current BHVs are susceptible to development of structural valve deterioration (SVD) which renders them incapable of recapitulating life-long function of native valve leaflets, particularly in younger patients due to their increased immune system activity [5].

Recent advances in antigen removal/decellularization have advanced the possibility of using unfixed xenogeneic extracellular matrix (ECM) scaffolds as heart valve leaflet biomaterials. ECM scaffolds provide an alluring alternative to glutaraldehyde-fixation, since they offer the potential to overcome recipient graft-specific immune responses, while simultaneously fostering non-immune cellular repopulation allowing for ECM turnover, repair, remodeling and growth [9]. The process of antigen removal/decellularization utilizes various chemical (e.g., detergents, reducing agents, enzymes) and mechanical (orbital shakers, perfusion, freeze-thaw cycles) approaches to target the removal of particular cellular components and/or groups of antigens, to reduce biomaterial antigenicity while maintaining native ECM structure and function [9–11]. However, although extensive studies have been conducted examining the static structure and function of antigen removed/decellularized ECM scaffolds, the extent to which candidate tissues are capable of withstanding cyclic mechanical loading experienced by heart valve leaflets remains unknown. Due to their homogeneous nature, ease of processing and single tissue type, bovine and porcine pericardia are currently the most predominantly studied unfixed heart valve leaflet biomaterials [5, 8, 12]. Determining which of these candidate tissues is most capable of withstanding cyclical heart valve leaflet loading conditions is critical to determining the optimal tissue type for production of unfixed ECM scaffold-based heart valve leaflets. Furthermore, the effect of cyclic loading on unfixed tissues must be determined before the balance between cycle dependent deterioration and recipient repopulating cell mediated remodeling in such biomaterials can be predicted.

The first step, therefore, in application of unfixed xenogeneic ECM scaffolds as heart valve leaflet biomaterials is to determine the effect of cyclic loading on candidate xenogeneic tissue structure, composition and function. Specifically, we hypothesized that unfixed xenogeneic tissues would undergo significantly greater cycle dependent strain, structural disruption, compositional alteration, and loss of mechanical integrity than current clinically utilized glutaraldehyde-fixed biomaterials. Consequently, in this manuscript we: 1. quantify the extent and time frame of cyclic dependent strain in unfixed-tissues (porcine pericardium and bovine pericardium) compared to fixed-tissue (glutaraldehyde-fixed bovine pericardium), and 2. identify the predominant structural, compositional, and functional mechanisms responsible for cycle dependent strain in each material.

## Materials and methods

Unless stated otherwise, all chemicals were purchased from Sigma-Aldrich, St. Louis, MO, USA. Expanded methods are available in supplementary material.

### Tissue harvest

All experimental procedures and protocols were approved by the Mayo Clinic Institutional Animal Care and Use Committee (IACUC) and performed in accordance with the relevant guidelines and regulations from the Guide for the Care and Use of Laboratory Animals [13]. Fresh bovine pericardium (BP) was harvested immediately postmortem from young adult cattle (Spear Products, Coopersburg, PA, USA), shipped on dry ice and stored at -80°C upon arrival. Porcine pericardium (PP) was harvested immediately postmortem and stored in Dulbecco’s Modified Eagles Medium (DMEM) with 15% (v/v) dimethyl sulfoxide (DMSO) at -80°C. Glutaraldehyde-fixed bovine pericardium (GFBP) patches were purchased from Abbot, formerly St. Jude Medical, (St. Paul, MN) and stored at RT in provided storage solution as per manufacturer’s instructions [14, 15].

### Heart valve tester

**Fig 1:**
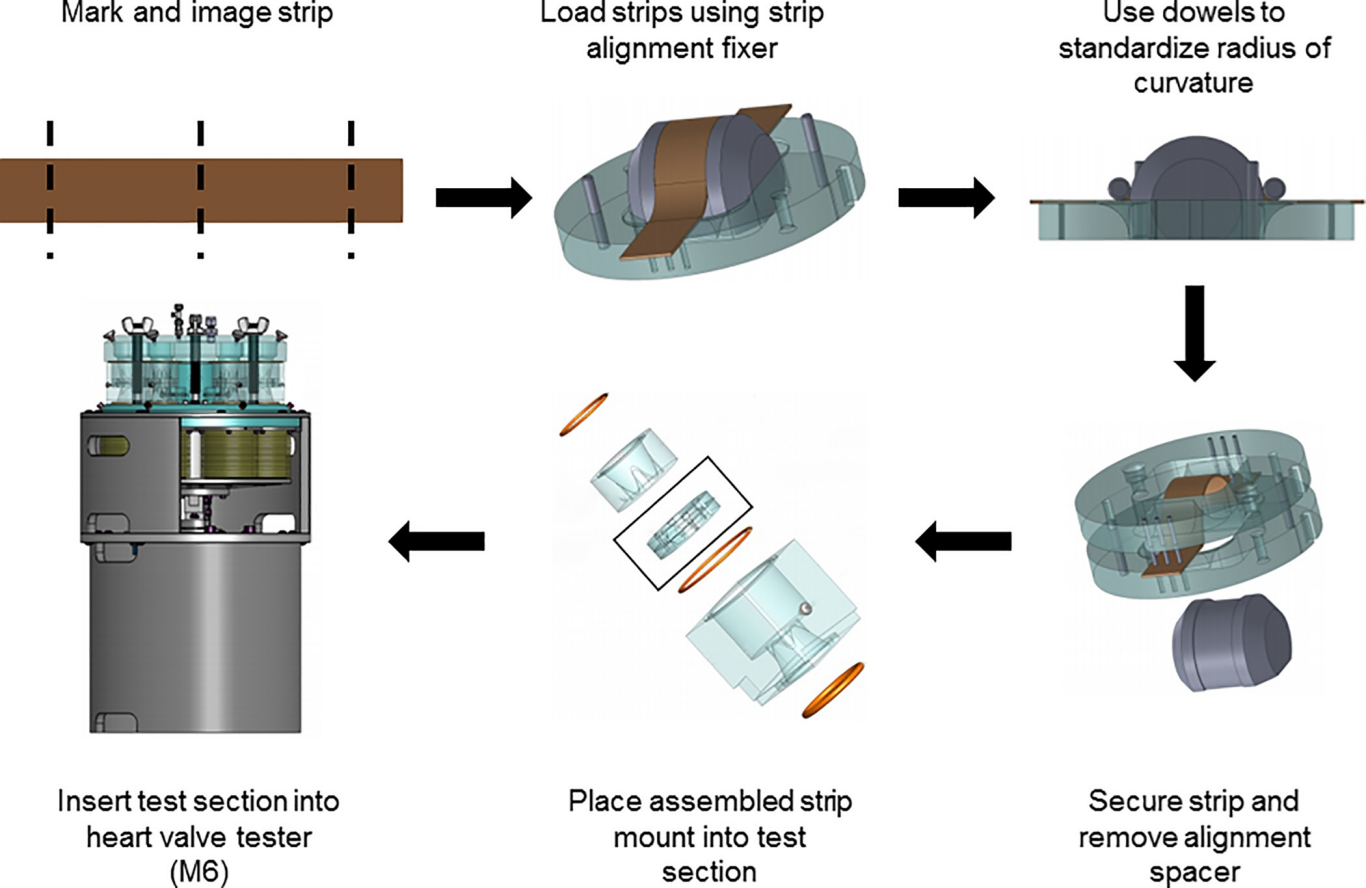
Flow diagram depicting how strips of pericardia were loaded to undergo consistent cyclic deformation. Tissues were harvested and cut into strips, marked and imaged. Strips were then loaded to maintain a standard radius of curvature in cyclic deformation and ran in Dynatek’s M6 Heart Valve Tester.

Porcine pericardium, bovine pericardium, and glutaraldehyde-fixed bovine pericardium were cut into 1 x 6 cm strips using a custom-made punch (Dynatek Labs, Galena, MO). The middle 4 cm of each strip was marked with 3 equally spaced lines, perpendicular to the long axis, using permanent ink composed primarily of alcohols and secondarily of ethylene glycol monobutyl ethers (Fig 1) [16]. Strips were photographed under zero strain. Once imaged, samples were loaded for strip accelerated wear testing on an M6 Heart Valve Tester (Dynatek, Galena, MO) according to the manufacturer’s instructions (Fig 1). The test sections were custom designed to ensure radius of curvature at maximal deformation was equivalent to that of native aortic valve cusps. All three groups (n = 2 per group) were run simultaneously at 37°C, 1400 cycles per minute (CPM) with an approximately sinusoidal waveform, and 120 mmHg in saline with 1% (v/v) antimycotic antibiotic solution (AAS) and 0.5mM Pefabloc. 1400 cycles per minute was determined to be the optimal acceleration whilst still maintaining physiologic pressure and boundaries. Both the 10 million cycles and 20 million cycles were run three times to result in a total of n = 6 per group and cycle number. All groups and analyses had n = 6 samples/group/cyclic loading number. Sample testing (e.g., biochemical analysis and strain) required each sample to be destroyed post-cycling, and therefore new samples were used to compare 0, 10, and 20 million cycles. Each sample was compared to its physiological control, and the percent difference between the two was then used to compared changes occurring between the 0, 10, and 20 million groups. The accelerated wear testing regime employed a relatively uniform load to the tissue (see supplementary material), such that during uniaxial tension the entire sample gauge region underwent relatively uniform fatigue. Upon completion of cycles, sections were removed from the M6 tester and the tissue strips carefully recovered from the section. Strips were re-imaged under zero strain and then stored in DMEM with 15% (v/v) DMSO at -80°C.

### Cycle dependent strain

Images of tissue pre- and post-cycling were uploaded to ImageJ, with strip width and distance between the two outer markings measured for each strip. The percent difference between pre- and post-cycle length was calculated and defined as the cycle dependent longitudinal strain each strip had undergone following cyclic deformation. Similarly, the pre- and post-cycle percentage difference in strip width was calculated and defined as the cycle dependent perpendicular strain (n = 6 per group, per cycle number) [15].

### Finite element modeling

**Fig 2:**
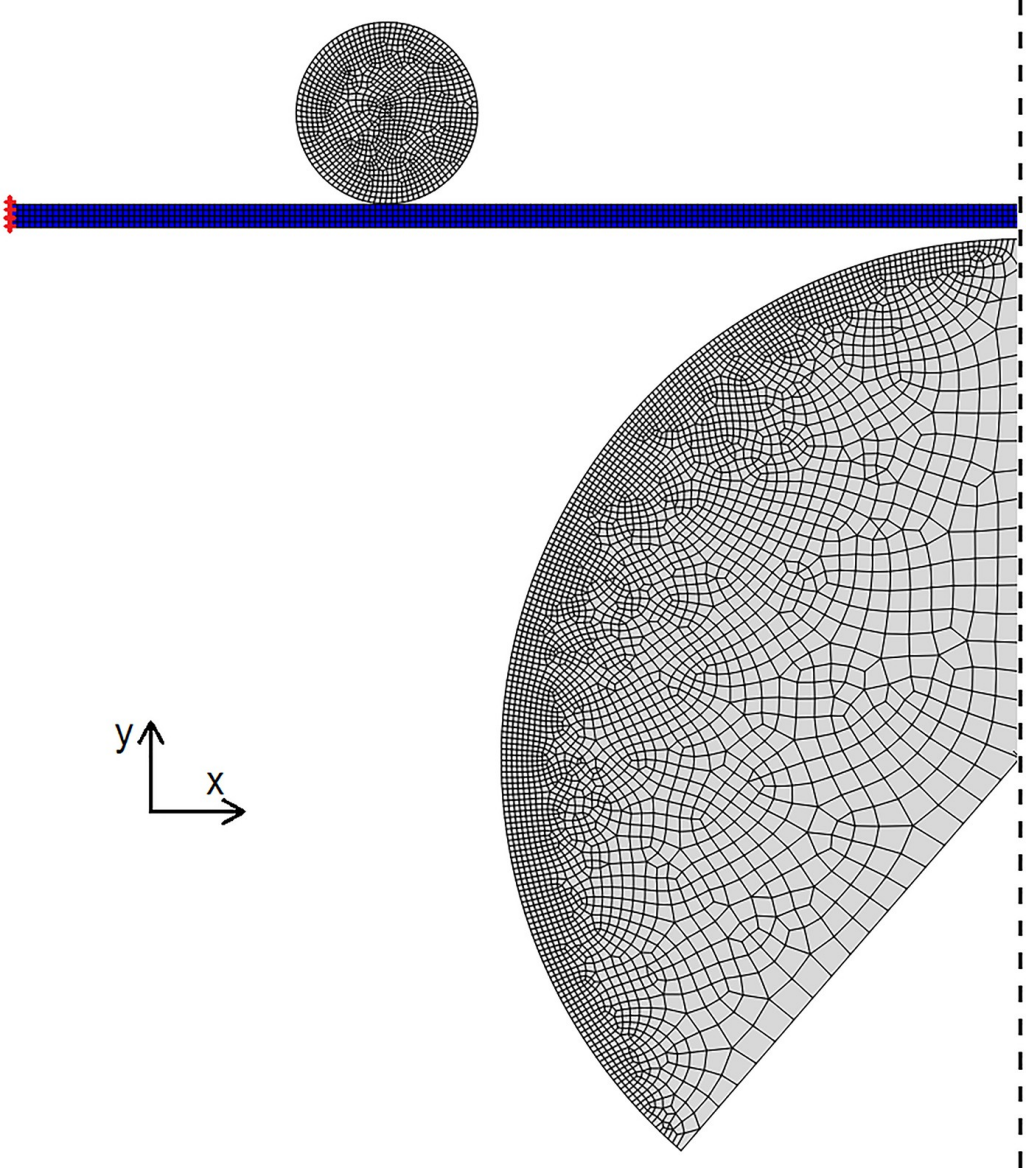
Finite element model of the testing procedure to investigate the stresses applied to the tissue and whether they are physiological. Continuum elements are in blue and rigid bodies are in grey. The dashed line indicates a plane of symmetry and the red markers indicate the region where displacement boundary conditions were applied. The simulation consisted of two steps; the first used the larger spherical rigid body to deform the sample to the radius of curvature at the start of the *in vitro* testing. The elements on the left edge highlighted were fixed in the y direction but could move in the x direction to allow the tissue to be pulled over the larger contact body. The second step removed this contact body then applied the maximum pressure in the *in vitro* testing (260 mmHg) to the base of the *in silico* sample. Here the highlighted elements were then held in both directions to mimic the sample being pinned.

To verify stresses applied in the heart valve tester were representative compared to previous studies, the experimental arrangement was replicated in a finite element (FE) model (Fig 2). The simulation consisted of two steps; the first used the larger spherical rigid body to deform the sample to the radius of curvature at the start of *in vitro* testing. The highlighted elements on the left edge were fixed in the y direction but could move in the x direction, allowing the tissue to be pulled over the larger contact body. The second step removed this contact body, then applied maximum pressure in the *in vitro* testing (260 mmHg) to the base of the *in silico* sample. Here the highlighted elements were then held in both directions to mimic the sample being pinned. Due to the uniform elongation of samples (see supplementary material) we assumed that loading had been relatively evenly applied. Therefore, plain strain conditions were assumed, and additionally, the pressure was assumed to have been applied evenly to the base of the sample. Symmetry was also applied to reduce simulation time. The tissue strip was meshed with 700 4-node quadrilateral hybrid formulation plane strain elements, with incompatible modes to aid with bending experienced during the simulation. The larger rigid body was meshed with 3529 plane strain elements while the smaller had 1046 plane strain elements. Contact was assumed frictionless and the model was constructed using ABAQUS and solved using the static implicit method. The tissue material properties were assumed isotropic and described using a 2^nd^ order polynomial strain energy function:

$$W=\sum_{i+j=1}^{2}C_{ij}{\left({\bar{I}}_{1}-3\right)}^{i}{\left({\bar{I}}_{2}-3\right)}^{j}+\sum_{i=1}^{2}\frac{1}{D_{i}}{\left(J-1\right)}^{2i}$$ (1)

with *C*_*ij*_ and *D*_*i*_ the material parameters and *J* the volume ratio. ${\bar{I}}_{1}={\bar{\lambda }}_{1}^{2}+{\bar{\lambda }}_{2}^{2}{+\bar{\lambda }}_{3}^{2}$ and ${{\bar{I}}_{2}=\bar{\lambda }}_{1}^{-2}+{\bar{\lambda }}_{2}^{-2}+{\bar{\lambda }}_{3}^{-2}$ are the first and second isochoric strain invariants where the isochoric stretches ${\bar{\lambda }}_{i}$ are given by ${\bar{\lambda }}_{i}=J^{-\frac{1}{3}}\lambda_{i}$ with *λ*_*i*_ the principal stretches. Material parameters were fit to the average glutaraldehyde-fixed bovine pericardium data with the fitting performed by ABAQUS. The coefficients found were: *C*_10_ = 2.01, *C*_20_ = −133.36, *C*_01_ = −1.85, *C*_11_ = 333.28 and *C*_02_ = −185.87 MPa with *D*_1_ = 0.12 MPa, and *D*_2_ = 0 MPa.

### Uniaxial tensile testing

To examine the effects of the fatigue on stiffness and failure strength, uniaxial tensile testing was performed. Biaxial loading would provide greater physiological relevance as it reflects the *in vivo* loading conditions more closely [17–19]. However, at the high loads present in this study there are practical difficulties associated with using biaxial testing which may yield poor estimates of stress [20–22]. Consequently, uniaxial tensile testing was considered to be better suited to the investigation of failure behavior in the current study.

As previously reported, samples were dog boned using a custom-made punch with gauge length of 4 mm and 2 mm width of the dog bone region. All samples were prepared for mechanical testing, with the dog bone oriented along the longitudinal aspect of the strip used in cyclic deformation experiments. Samples were mounted between grips under zero strain and subjected to a 5% gauge length/sec strain rate (Instron Model 5565, Canton, MA). Thickness and width of dog boned samples were calculated from digital images imported into ImageJ (Wayne Rasband, National Institutes of Health, USA). Nominal stress was calculated from

$$\sigma_{nom}=\frac{F}{wT}$$ (2)

where *F* is the measured force, *w* is the width of the dog bone region, and *T* the sample thickness. Strain was found by taking the ratio of the machine head displacement to the original sample length. The ultimate tensile strength (UTS) and the tangent modulus in the linear region where then found from the resulting stress strain data (n = 6 per group, per cycle) [14, 15].

### Quantitative biochemistry

For all biochemical analyses, 6 mm discs of tissue were weighed, lyophilized for 72 h and re-weighed. Tissue hydration was assessed by calculating the percent loss of sample mass following lyophilization (n = 6 per group, per cycle) [14]. Samples underwent oxalic acid digestion and were assessed for elastin content using Fastin Elastin assay (Bicolor Ltd., Carrickfergus, UK). Samples underwent hydrochloric acid digestion and were assessed for collagen content using Hydroxyproline assay (Chondrex Inc., Redmond, WA) (n = 6 per group/per cycle) [14, 15].

### Histology

3 mm biopsy punches from samples (*n* = 6 per group, per cycle) were subjected to formalin-fixation and paraffin embedding. Hematoxylin and Eosin (H&E) staining for ECM morphology and nuclei visualization. Verhoeff van Gieson staining (VVG) and Picro-Sirius Red (PSR) staining were utilized for assessment of elastin and collagen organization respectively. Birefringence images were obtained using polarized light microscopy images of representative high-power fields (400x) from PSR stained sections, throughout the full thickness of the tissue (from the serous parietal surface to the fibrous adventitial surface of the pericardium). Birefringence was quantified using limit-to-threshold within ImageJ to determine the percent area of collagen alignment. All images were taken at 200x and 400x magnification using Nikon Eclipse Ni-E microscope (Nikon, Melville, NY) [15].

### Statistical analysis

All data are expressed as mean ± standard deviation (SD), and were analyzed using two-way analysis of variance. Tukey HSD post-hoc test and statistical significance defined at p<0.05.

## Results

### Finite element modeling

**Fig 3:**
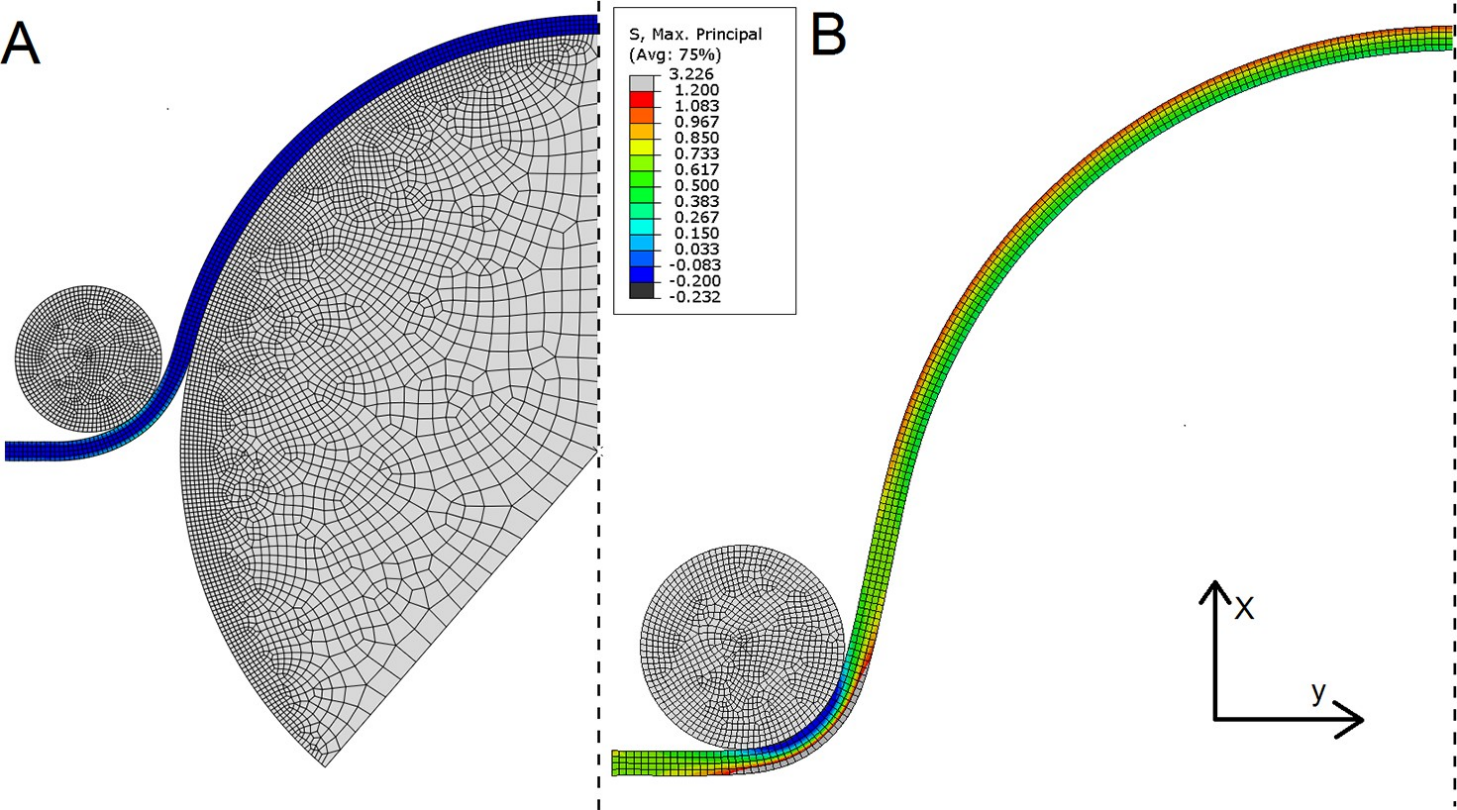
**Finite element model of the testing procedure at the end of the first step (A) and the end of the second step (B). For A, the tissue deformed around the two contact bodies with the profile of the tissue resembling that in the *in vitro* test. While for B, the tissue deformation exceeds that originally applied by the larger contact body and the maximum principal stress is also higher than at the end of the first step.** As expected, the maximum principal stress is far lower in A than B. In the second step (B), the maximum principal stress in the central region of the model where the largest deflection occurred was 1.2 MPa and was located on the upper side of the tissue. The lower maximum principal stress on the underside of the tissue was approximately 0.42 MPa. The stress distribution is relatively consistent along the tissue likely because of the uniform loading applied.

FE modelling was utilized to verify whether the stresses applied to the tissue by the heart valve tester were indeed physiological (Fig 2). The end of the first step shows the tissue deforming around the two contact bodies with the shape of the tissue resembling that in the *in vitro* test (Fig 3A). In the second step the tissue deformation exceeds that originally applied by the larger contact body and that the stress is also higher than at the end of the first step (Fig 3B). The maximum principal stress in the central region of the model where the largest deflection occurred was 1.2 MPa and was located on the upper side of the tissue. The lower maximum principal stress on the underside of the tissue was approximately 0.42 MPa. The stress distribution was relatively consistent along the tissue until the clamped region by the contact body, where the stress was greatest.

### Cycle dependent strain

**Fig 4:**
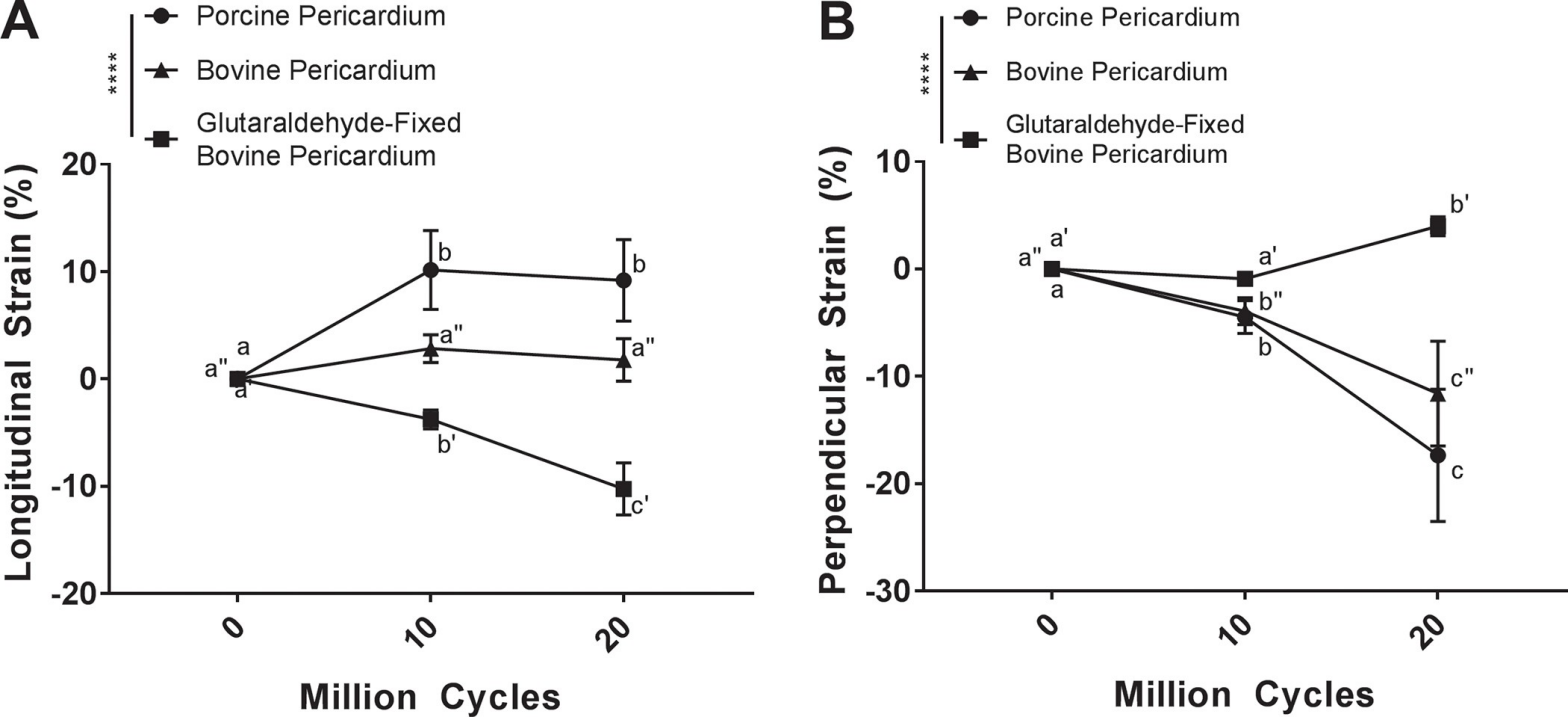
Cyclic dependent strain is dependent on tissue source and fixation. Longitudinal cycle dependent strain did not occur in unfixed bovine pericardium (A). Conversely, glutaraldehyde-fixed bovine pericardium exhibited negative longitudinal strain with increased cycle number (A). Porcine pericardium exhibited positive cycle dependent longitudinal strain which plateaued by 10 million cycles (A). Both bovine and porcine pericardium underwent negative cycle dependent perpendicular strain with increased cycle number (B). Glutaraldehyde-fixed bovine pericardium underwent positive cycle dependent perpendicular strain at 20 million cycles (B). Samples were assessed using a two-way analysis of variance, comparing means between tissue types and cycle number. n = 6 per group, per cycle number. Groups not connected by the same lower case letter (porcine pericardium), lower case double apostrophe (bovine pericardium), or lower case single apostrophe (glutaraldehyde-fixed bovine pericardium) are statistically significantly different. Data represent the mean ± s.d.

To determine the effect of cyclic deformation on potential heart valve biomaterials, cycle dependent longitudinal and perpendicular strain was quantified following strip configuration accelerated wear testing (Fig 1). Comparing the measured changes in dimensions, porcine pericardium underwent longitudinal elongation from baseline to 10 million cycles (10.16 ± 3.67%) (p<0.0001). Although the elongation was statistically significant between baseline and 20 million cycles (9.55 ± 4.12%) (p<0.0001), elongation plateaued at 10 million cycles and there was no difference between 10 and 20 million cycles (p = 0.7506) (Fig 4A). Porcine pericardium underwent perpendicular contraction over 10 million (-4.47 ± 1.54%) and 20 million cycles (-17.35 ± 6.16%) when compared to baseline (p = 0.0035 and p<0.0001 respectively). Furthermore, 20 million cycles resulted in greater perpendicular contraction than at 10 million cycles (p<0.0001) (Fig 4B). No statistically significant longitudinal elongation was observed for bovine pericardium over 10 million (2.83 ± 1.29%) or 20 million cycles (2.62 ± 0.77%) when compared to baseline (p = 0.1025 and p = 0.3460 respectively), or between cycles (p = 0.7346) (Fig 4A). However, bovine pericardium underwent perpendicular contraction at 10 million (-4.18 ± 1.23%) and 20 million cycles (-11.60 ± 4.88%) compared with baseline (p = 0.0334 and p<0.0001 respectively) as well as between cycles (p<0.0001) (Fig 4B). By comparison, glutaraldehyde-fixed bovine pericardium demonstrated longitudinal contraction at both 10 million (-3.76 ± 0.88%) and 20 million cycles (-10.25 ± 2.43%) compared to baseline (p = 0.0215 and p<0.0001 respectively). This longitudinal contraction increased between 10 and 20 million cycles (p<0.0001) (Fig 4A). Perpendicular elongation of glutaraldehyde-fixed bovine pericardium samples increased from baseline following 20 million (4.00 ± 0.88%) but had no statistically significant change after 10 million cycles (-0.90 ± 0.59%) (p = 0.0299 and p = 0.8223 respectively). Perpendicular elongation also increased between 10 and 20 million cycles (p = 0.0063) (Fig 4B).

### Mechanical testing

**Fig 5:**
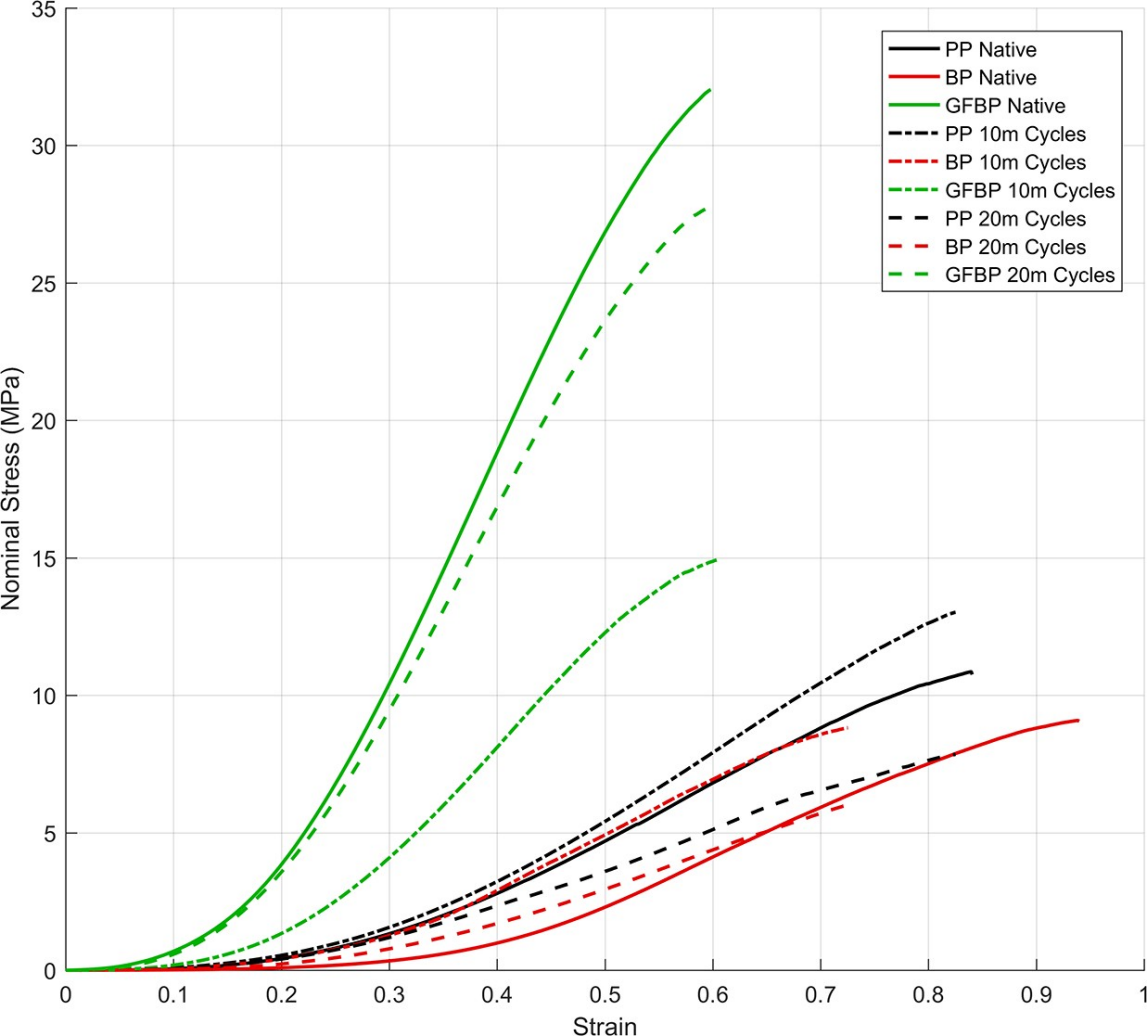
Nominal stress versus strain curve of native tissue, and tissue that has undergone 10 million (10m) or 20 million (20m) cycles of loading in the heart valve tester. Tissue types were porcine pericardium (PP), bovine pericardium (BP), or glutaraldehyde fixed bovine pericardium (GFBP). Overall despite the differences between tissue types and fatigue the curves show a similar trend of initial strain-stiffening followed by a linear region and then a decrease in gradient to breaking. This may be interpreted as collagen fibers unfurling then becoming stretched before gradually damaging and ultimate failure.

**Fig 6:**
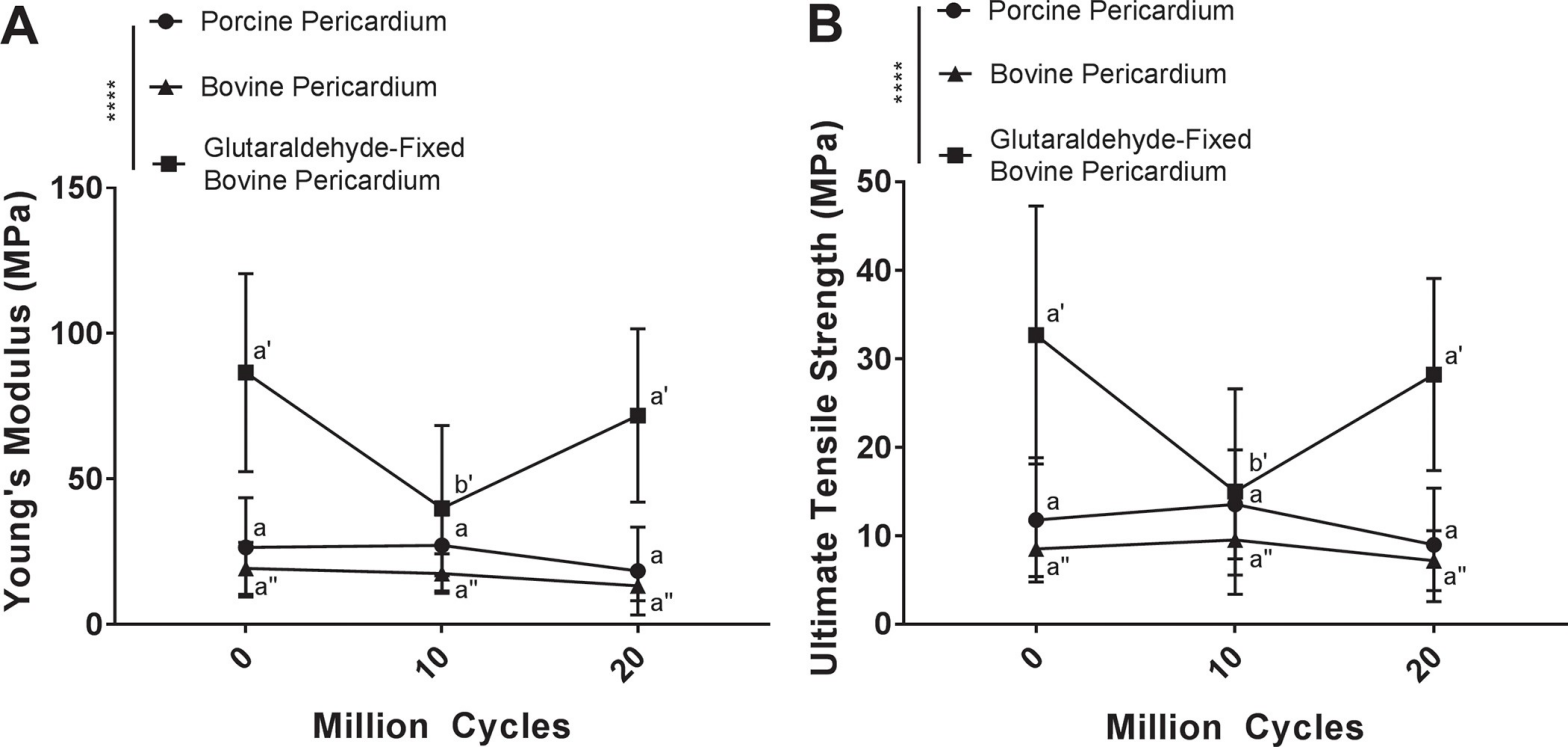
Cyclic deformation alters mechanical properties of glutaraldehyde-fixed bovine pericardium, but leaves porcine pericardium and bovine pericardium unchanged. Tangent modulus (A) and ultimate tensile strength (B) of glutaraldehyde-fixed bovine pericardium decreased following 10 million cycles of deformation but returned to baseline levels at 20 million cycles. No alteration in tangent modulus or ultimate tensile strength of either porcine or bovine pericardium was found following cyclic deformation. Tangent modulus and ultimate tensile strength were assessed using a two-way analysis of variance, comparing means between tissue types and cycle number. n = 6 per group, per cycle number. Groups not connected by the same lower case letter (porcine pericardium), lower case double apostrophe (bovine pericardium), or lower case single apostrophe (glutaraldehyde-fixed bovine pericardium) are statistically significantly different. Data represent the mean ± s.d.

Effect of cyclic deformation on mechanical properties of potential heart valve biomaterials was assessed using uniaxial tensile testing. Overall despite the differences between tissue types and fatigue, the curves show a similar trend of initial strain-stiffening followed by a linear region and then a decrease in gradient to breaking (Fig 5). However, the magnitudes of the UTS and modulus are different. This may be interpreted as collagen fibers unfurling then becoming stretched before gradually damaging and ultimate failure. Comparing the tangent modulus in the linear region and the UTS, for porcine pericardium, no statistically significant change in tangent modulus occurred between baseline (26.45 ± 17.07 MPa) 10 million (27.12 ± 10.48 MPa) or 20 million cycles (18.31 ± 15.13 MPa) (p = 0.9960 and p = 0.5757 respectively) (Fig 6A). Similarly, no statistically significant difference in UTS compared to baseline (10.86 ± 6.33 MPa) was found following 10 million (13.02 ± 6.32 MPa) or 20 million cycles (7.85 ± 5.10 MPa) (p = 0.8445 and p = 0.6820 respectively) (Fig 6B). Bovine pericardium demonstrated no significant difference in tangent modulus following 10 million (15.91 ± 8.22 MPa) or 20 million cycles (12.95 ± 5.27 MPa) when compared to baseline (19.26 ± 8.92 MPa) (p = 0.9732 and p = 0.7146 respectively) (Fig 6A). Similarly, no statistically significant change in UTS occurred compared to baseline (9.09 ± 4.52 MPa) following 10 million (8.82 ± 4.33 MPa) or 20 million cycles (6.01 ± 3.13 MPa) (p = 0.9524 and p = 0.9093 respectively) (Fig 6B). Tangent modulus of glutaraldehyde-fixed bovine pericardium decreased following 10 million cycles (39.93 ± 28.42 MPa) compared to baseline (86.53 ± 34.00 MPa) (p<0.0001) (Fig 6A). Interestingly, tangent modulus returned to baseline levels by 20 million cycles (71.82 ± 29.79) (p = 0.1266) (Fig 6A). The UTS of glutaraldehyde-fixed bovine pericardium showed a similar trend as tangent Modulus, with a decrease in UTS at 10 million cycles (14.92 ± 10.95 MPa) compared to baseline (32.05 ± 11.60 MPa) (p<0.0001), and a return to baseline levels following 20 million cycles (27.84 ± 12.75 MPa) (p = 0.3547) (Fig 6B).

### Biochemical analysis

**Fig 7:**
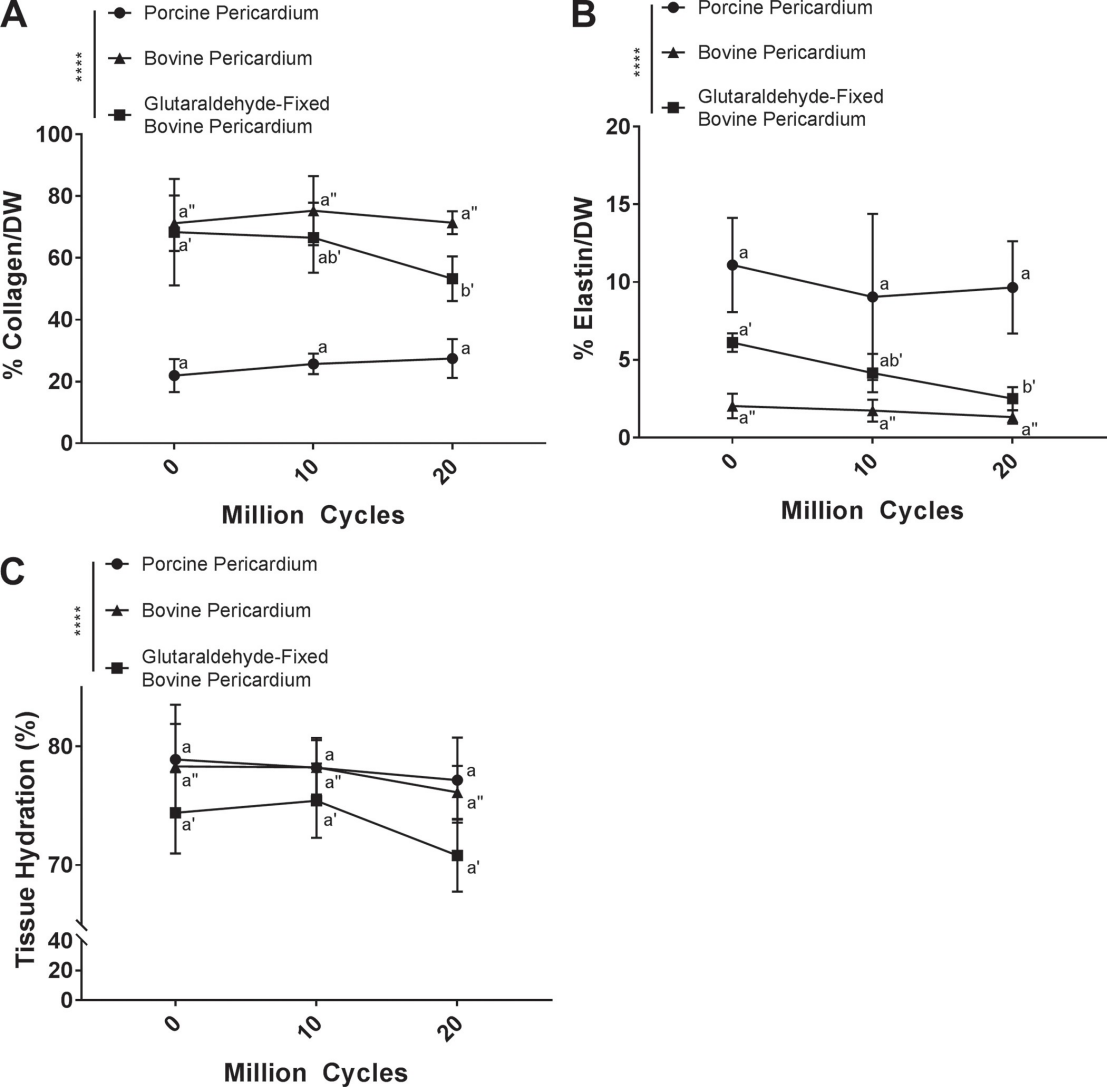
Biochemical composition of glutaraldehyde-fixed bovine pericardium is altered following cyclic deformation, but maintained in porcine pericardium and bovine pericardium. Both collagen (A) and elastin (B) content of glutaraldehyde-fixed bovine pericardium decreased following 20 million cycles. Biochemical composition of both porcine and bovine pericardium was unaltered by cyclic deformation. There was no change in tissue hydration with increased cycle number for any of the tissues (C). Biochemical composition was assessed using a two-way analysis of variance and comparing means between tissue types and cycle number. n = 6 per group, per cycle number. Groups not connected by the same lower case letter (porcine pericardium), lower case double apostrophe (bovine pericardium), or lower case single apostrophe (glutaraldehyde-fixed bovine pericardium) are statistically significantly different. Data represent the mean ± s.d.

Changes in composition of tissue undergoing cyclic deformation was assessed by quantification of collagen content, elastin content, and tissue hydration. Porcine pericardium had no statistically significant change in collagen content following 10 million (25.69 ± 3.31%) or 20 million cycles (27.46 ± 6.27%) compared to baseline (21.92 ± 5.35%) (p = 0.7563 and p = 0.5500 respectively) (Fig 7A). Similarly, bovine pericardium demonstrated no statistically significant change in collagen content following 10 million (75.28 ± 11.18%) and 20 million cycles (71.40 ± 3.69%) compared to baseline (71.22 ± 9.00%) (p = 0.7233 and p = 0.9994 respectively) (Fig 7A). Conversely, for glutaraldehyde-fixed bovine pericardium, cyclic deformation resulted in a reduction in collagen content following 20 million cycles (50.48 ± 2.76%) compared to baseline (68.32 ± 17.23%) (p = 0.0127), although collagen content was not different from baseline at 10 million cycles (66.53 ± 11.31%) (p = 0.9347) (Fig 7A).

Elastin content of porcine pericardium was maintained following both 10 million (6.94 ± 1.47%) and 20 million cycles (9.66 ± 2.97%), compared to baseline (11.10 ± 3.03%) (p = 0.1858 and p = 0.4283 respectively) (Fig 7B). Similarly, bovine pericardium also maintained elastin content following 10 million (1.74 ± 0.71%) and 20 million cycles (1.33 ± 0.43%) compared to baseline (2.04 ± 0.78%) (p = 0.9623 and p = 0.8063 respectively) (Fig 7B). Conversely, elastin content of glutaraldehyde-fixed bovine pericardium decreased with increasing cycle number, reaching statistical significance following 20 million cycles (2.51 ± 0.74%) compared to baseline (6.12 ± 0.60%) (p = 0.0144) (Fig 7B).

Hydration of porcine pericardium was maintained following 10 million (76.78 ± 4.16%) and 20 million cycles (77.17 ± 3.58%), compared to baseline (78.90 ± 4.61%) (p = 0.09263 and p = 0.5878 respectively) (Fig 7C). Similarly, hydration of bovine pericardium was not significantly changed following 10 million (78.24 ± 2.30%) and 20 million cycles (76.13 ± 2.24%) compared to baseline (78.31 ± 3.59%) (p = 0.9990, p = 0.4213 respectively) (Fig 7C). Glutaraldehyde-fixed bovine pericardium showed a slight decrease in hydration following 20 million cycles (70.80 ± 3.04%) compared to both baseline (74.40 ±± 3.43%) and 10 million cycles (75.43 ± 3.13%), however this finding failed to reach statistically significance (p = 0.0961 and p = 0.0565 respectively) (Fig 7C).

### Histology

**Fig 8:**
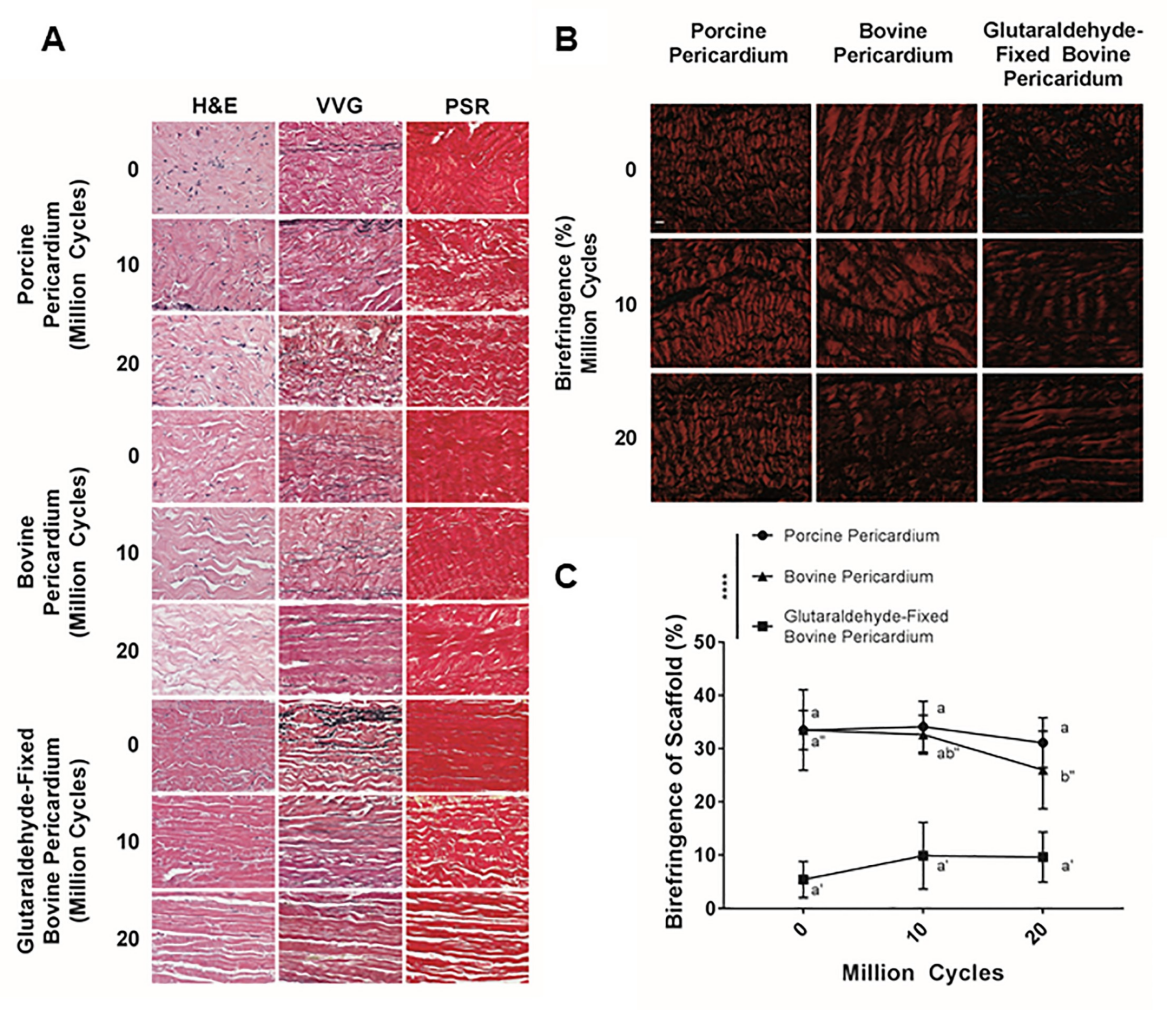
Qualitative histological assessment of fixed and unfixed tissue biomaterials following cyclic deformation. Nuclei density was maintained across all groups regardless of cycle number (hematoxylin and eosin (H&E)) (A). Elastin content decreased with increasing number of cycles for glutaraldehyde-fixed bovine pericardium, whereas both porcine and bovine pericardium maintained elastin content (Verhoeff-Van Gieson (VVG)) (A). However, elastin in porcine pericardium appeared fragmented with increasing number of cycles. All three tissue types underwent some cycle dependent separation of collagen bundles, although this was more dramatic in porcine pericardium and glutaraldehyde-fixed bovine pericardium than in bovine pericardium. n = 6 per group, per cycle number. Scale bar represents 50 μm (A). Collagen birefringence (B) of glutaraldehyde-fixed bovine pericardium at baseline is less than that of either of the non-fixed tissues (C). Collagen birefringence was significantly reduced following 20 million cycles for bovine pericardium (C). Glutaraldehyde-fixed bovine pericardium exhibited a trend towards increased collagen birefringence with increased cycle number, although this finding failed to reach significance (C). Percentage of birefringence was assessed using a two-way analysis of variance, comparing means between tissue types and cycle number. Scale bar represents 10 μm. n = 6 per group, per cycle number. Groups not connected by the same lower-case letter (porcine pericardium), lower-case double apostrophe (bovine pericardium), or lower-case single apostrophe (glutaraldehyde-fixed bovine pericardium) are statistically significantly different. Data represent the mean ± s.d.

Changes in structure of tissue undergoing cyclic deformation were qualitatively and semi-quantitatively assessed using histology and collagen birefringence respectively. Presence of cell nuclei assessed on H&E staining was unchanged for all groups regardless of cycling (Fig 8A). For porcine pericardium, although elastin content was maintained at 10 and 20 million cycles compared to baseline, fragmentation of elastin increased with number of cycles (Fig 8A). No changes in bovine pericardial histologic appearance were identified between baseline and cycled samples (Fig 8A). For glutaraldehyde-fixed bovine pericardium, elastin content decreased with increasing number of cycles (Fig 8A). Furthermore, glutaraldehyde-fixed bovine pericardium collagen content and cohesiveness of collagen bundles (i.e., size and number of void spaces between bundles) decreased with increasing cycle number (Fig 8A).

Using polarized light (Fig 8B), no statistically significant change in collagen birefringence was found for porcine pericardium undergoing 10 million (34.12 ± 4.78%) or 20 million cycles (31.11 ± 4.67%) compared to baseline (33.49 ± 7.56%) (p = 0.9685 and p = 0.6422 respectively) (Fig 8C). Collagen birefringence of bovine pericardium decreased with increasing cycle number, reaching statistical significance at 20 million cycles (26.00 ± 7.29%) compared to baseline (33.49 ± 3.67%) (p = 0.0263) (Fig 8C). Glutaraldehyde-fixed bovine pericardium showed a non-statistically significant trend towards increasing in birefringence between 10 million (9.91 ± 6.28%) and 20 million cycles (9.65 ± 4.71%) compared to baseline (5.44 ± 3.39%) (p = 0.2260 and p = 0.2651 respectively) (Fig 8C).

## Discussion

A critical step in translation of unfixed-tissue for use in bioprosthetic heart valves, is to understand the effect of cyclic loading on candidate tissue types. These steps both aid in starting tissue selection and in identifying changes in tissue response to cyclic loading, which may occur as part any antigen removal/decellularization processes applied to the tissue prior to valve fabrication. The current work demonstrates that unfixed bovine pericardium undergoes significantly less cyclic dependent strain than either unfixed porcine pericardium or glutaraldehyde-fixed bovine pericardium. Specifically, the results demonstrate that unlike other candidate tissues tested, unfixed bovine pericardium maintains native structure, composition and function over 20 million cycles (i.e., equivalent to 6 months of *in vivo* performance) of accelerated wear testing. Consequently, we conclude that unfixed bovine pericardium has potentially ideal cyclic loading properties for formation of biological heart valve leaflets.

Cellular repopulation, either *in vitro* or *in vivo*, of unfixed xenogeneic biomaterials for regenerative medicine applications, is expected to result in ECM turnover and biomaterial replacement with recipient tissue [9, 23]. Although the time-course for such ECM remodeling and replacement may vary dependent on the decellularization/antigen removal process utilized, available reports indicate the process may be completed in 6–12 months [9, 23–26]. Consequently, unlike current glutaraldehyde-fixed bioprostheses, the design criteria for resistance of cyclic deformation for an unfixed xenogeneic heart valve leaflet are likely to only extend to approximately 6 months of *in vivo* performance. By this time point, such biomaterials would be expected to be largely or completely remodeled by repopulating cells. Therefore, this study focused on assessing the effect of 10 and 20 million cycles of cyclic deformation rather than utilizing the 200 million cycles detailed in ISO5840 for FDA approval of current glutaraldehyde-fixed heart valve biomaterials [7].

Although the full valvular geometry of standard accelerated wear testing is more physiologically relevant, it was also less appropriate for analysis as the location of stress concentrations are relatively unknown and vary across the sample. Consequently, for tensile testing, it is unknown whether the cut sample is from the most fatigued region of tissue and whether the sample has fatigued uniformly, resulting in varying strength over gauge region. Similarly, fatigue testing performed by tensile test equipment cannot operate at similarly high frequencies as our accelerated wear tester. As such, samples will take far longer to test, making the testing modality less physiologically relevant. To verify the fatigue testing effectively reproduced maximum stresses applied *in vivo*, FE modelling was used to replicate *in silico* testing conditions. The stresses in our model were relatively similar when compared to previous work giving confidence that the testing applied here sufficiently replicated *in vivo* conditions, despite the relatively dissimilar loading behavior, and that the changes to the tissues is due to fatigue from the cyclic loading [27–33]. Similar to Noble et al. [34], we utilized an isotropic polynomial strain energy function and a plane strain FE model to emulate the experimental conditions of an anisotropic material loaded primarily along one of its axes. More complex strain energy functions, capable of describing a wider range of material responses (including, for example, anisotropic effects) are available: two such models, with differing phenomenological backgrounds, are described in Gasser et al. 2006 or Bischoff et al. 2002 for example [35, 36]. However, these provide no advantage in the present scenario; the sample is only significantly loaded along one axis and there is relatively small out of plane loading. Thus, if a 3D model and an anisotropic constitutive model were utilized this additional complexity would be describing an axis in which there is no loading and would be redundant for the purposes of this simulation.

Glutaraldehyde-fixed bovine pericardium remains the most popularly utilized bioprosthetic heart valve leaflet biomaterial in clinical application, making it the clinical gold standard [1]. The fixed biomaterials utilized in such valves have met the cycle dependent strain criteria set forth by the FDA of 200 million cycles in an accelerated wear tester operating at pressures across the valve ranging between 90 and 120 mmHg at an opening/closing cycle rate of 13–25 Hz [7]. Furthermore, *in vivo* freedom from structural valve disease is approximately 10 years for such valves, suggesting that results obtained from accelerated wear testing have clinical relevance [4]. We therefore hypothesized that glutaraldehyde-fixed bovine pericardium used as a control in this study, would undergo minimal cyclic dependent strain compared to either of the unfixed pericardial tissues assessed. Surprisingly, the data demonstrated that glutaraldehyde-fixed bovine pericardium exhibited significant negative longitudinal strain following cyclic deformation, which peaked at –10% following 20 million cycles. In addition to straining longitudinally, glutaraldehyde-fixed bovine pericardium also demonstrated a 4% positive perpendicular strain at 20 million cycles. To the author’s knowledge, this is the first report of cycle dependent plastic deformation for such glutaraldehyde-fixed heart valve biomaterials. Previous reports detailing the immediate effects of glutaraldehyde fixation indicate that specifics of the fixative process (e.g., free vs tethered), material orientation (e.g., circumferential vs longitudinal) and pressures (e.g., high vs low) may alter the material’s mechanical properties by cross-linking elastin and collagen fibers [37–39]. Specifically, pressure fixation has been associated with variable degrees of tissue shrinkage and flattening of collagen crimping depending on the stress imposed [37, 40]. Although the fixative process of the material assessed in this manuscript is proprietary, it is known to be fixed under low pressure and as expected pre-cycling histology images demonstrate flattening of the collagen crimps. The process of fixation, even under low pressure, imposes a pre-stress on the material [37–40]. The observed negative longitudinal cycle dependent strain of glutaraldehyde-fixed bovine pericardium is likely due to release of the pre-stress imposed by the pressure fixation process, allowing the material to contract. This potential mechanism is supported by the alterations in the structure (i.e., increased separation of collagen bundles, and a trend towards increased alignment) of glutaraldehyde-fixed bovine pericardium which occur in response to cyclic loading [37–39, 41]. Furthermore, our results also show a substantial drop in the ultimate tensile strength of glutaraldehyde-fixed bovine pericardium at 10 million cycles compared to baseline, again supporting alterations in the materials structure and composition (i.e., reduced collagen and elastin content) as being instrumental to the observed cycle dependent strain. Surprisingly, the mechanical properties of glutaraldehyde-fixed bovine pericardium were found to return to baseline levels following 20 million cycles, despite continued structural and compositional deterioration. The process of glutaraldehyde fixation crosslinks collagen fibers, increasing the tangent modulus and ultimate tensile strength of the material compared to unfixed tissue [12, 41, 42]. However, as the material experiences cyclic dependent strain, crosslinks begin to fail, and the structure is altered. Therefore, the decrease in ultimate tensile strength witnessed at 10 million cycles is most likely due to inter-fiber crosslinks failing and altering the structural mechanics of the tissue. An additional and counteracting effect of collagen crosslinking has also been previously reported, namely inter-fiber crosslinks induce fiber immobility and thereby prevent collagen fiber realignment in response to stress [43]. The ability for collagen fibers to reorient and align along the lines of stress in uniaxial testing is crucial. Previous literature has demonstrated that, when inter-fiber crosslinks fail, greater fiber mobility is allowed [43]. Therefore, as the cycle number increases beyond 10 million and better fiber mobility is achieved, collagen fibers align along the plane of stress. This change in collagen fiber realignment may be responsible for the increased mechanical properties of the biomaterial observed at 20 million cycles. Additionally, significant decreases in both collagen and elastin content were evident following 20 million cycles. Previous reports have indicated that elastin limits the degree of reorientation in collagen alignment under both low and high strain [43]. Consequently, loss of elastin in glutaraldehyde-fixed bovine pericardium may further facilitate strain dependent reorientation of collagen fibers, accounting for the increased mechanical properties at 20 million cycles. Although not statistically relevant, the trend towards increased birefringence in glutaraldehyde-fixed bovine pericardium with increasing cycle number, may also be a reflection of cyclic deformation inducing increased collagen fiber orientation [44]. The complex interactions of these opposing compositional and structural changes may explain the bimodal alterations in mechanical properties of glutaraldehyde-fixed bovine pericardium. The cyclic dependent strain and subsequent changes in glutaraldehyde-fixed bovine pericardial structure and function demonstrated here may help elucidate the poorly understood mechanisms contributing to structural valve deterioration.

Porcine pericardium has also been investigated as a possible unfixed xenogeneic heart valve leaflet biomaterial [5, 8, 12]. Previous reports have shown that porcine pericardium has mechanical properties which meet or exceed those of native heart valve leaflets, thereby making it a candidate material for use in heart valve replacements [8, 45]. However, following cyclic deformation, porcine pericardium showed substantial positive longitudinal strain, plateauing at 10.16% following 10 million cycles (equivalent of 3 months *in vivo*). More so, the tissue demonstrated negative perpendicular strain, progressively thinning with increased number of cycles, which reached -17.35% after 20 million cycles. Such early cycle dependent deformation of the biomaterial is undesirable for heart valve leaflet applications, as it would be expected to lead to alterations in both radial and circumferential leaflet geometry over a time frame in which full tissue remodeling is unlikely to have occurred (i.e., 6–12 months) [23, 24]. Interestingly, the uniaxial tensile strength and tangent modulus of porcine pericardium were unchanged despite the observed cycle dependent deformation, with values for both parameters remaining similar to those previously reported for uncycled porcine pericardium [5, 8, 12]. This suggests that the observed plastic deformation is not caused by compositional alterations, but rather by alterations in material structure. Indeed, no changes in biochemical composition of collagen or elastin were identified in the current study. However, although elastin content is maintained, it appeared that the elastin alignment was altered following cyclic deformation, which could allow for the lengthening and thinning of the material, while still maintaining tangent modulus and ultimate tensile strength. Even though the static strength from uniaxial tensile testing may indicate that porcine pericardium is a suitable candidate for heart valve prostheses, the cycle dependent strain exhibited by the material suggests otherwise and should be considered in determining future application of porcine pericardium as an unfixed heart valve leaflet biomaterial. Both uniaxial tension testing and fatigue testing were performed on a single axis. For GFBP this is the only practical approach to the material since it undergoes fixation under pressure, which changes the surface patterning of the tissue. Bovine and porcine pericardium by comparison may be anisotropic but it is difficult to determine the fiber orientation non-destructively [46]. Additionally, the extent to which preferred fiber direction at the surface of pericardial tissues reflects fiber alignment throughout the full thickness of the material is unclear. Thus, samples were tested with random orientation, which increased variability between samples; however, as we found significance this assumption was deemed acceptable.

Bovine pericardium is currently the most popularly unfixed biomaterial investigated in decellularization/antigen removal approaches for heart valve tissue engineering [12]. This popularity may in part stem from the historical use of glutaraldehyde-fixed bovine pericardium in heart valve bioprostheses, but also likely relates to the relative ease of use of the material compared to porcine tissue. In the current study, unfixed bovine pericardium experienced no longitudinal strain as cycle number increased. Although the material slightly thinned by -11.6% (negative perpendicular strain) at 20 million cycles, this negative strain did not alter the mechanical properties of the tissue (i.e., ultimate tensile strength and tangent modulus). These findings are in accordance with previous non-cyclic studies, which demonstrated that bovine pericardium has a lower tangent modulus and ultimate tensile strength than porcine pericardium [12, 45]. Bovine pericardium also largely maintained its native structure and composition following cyclic deformation, although collagen alignment was mildly reduced by 20 million cycles. This finding may indicate that collagen fibrillary structure of bovine pericardium begins to deteriorate by 20 million cycles. However, since no longitudinal strain or deterioration in mechanical properties are present at 20 million cycles, this slight alteration in collagen alignment may be tolerable due to the anticipated cellular repopulation and matrix turnover in an *in vivo* implantation [9, 23, 24]. Indeed, previous work with bovine pericardium-based ECM scaffolds has shown complete cellular repopulation, evidence of collagen turnover and neovascularization within 42 days of implantation [47]. Therefore, the changes in collagen alignment seen *in vitro* may be counteracted by the development of constructive host cell repopulation and remodeling of such unfixed tissues. This concept highlights a less commonly discussed notion that cycles occurring *in vivo* with an unfixed-tissue scaffold could result in substantially more positive outcomes than cycles occurring *in vitro*. The previously demonstrated remodeling and adaptation of bovine pericardial ECM scaffolds occurring *in vivo* has potential to result in stress-adaptation and tissue remodeling to adapt to the physiologic environment [9, 26, 48]. Since *in vitro* cyclic deformation assumes an inert relationship between the biomaterial and its surroundings, it fails to account for such host-related responses and therefore may underestimate how the tissue performs at 6 months *in vivo*. Although the FDA currently dictates that bioprosthetic heart valves must remain functional following 200 million (or 5 years) in an accelerated wear tester, this criteria is unlikely to be applicable to unfixed-tissue biomaterials [7]. The remodeling and development of collagen previously reported for bovine pericardium at 6 months *in vivo* indicates that unfixed tissue is being integrated into the host tissue and remodeled [23, 49]. Therefore, current FDA criteria may need to be altered to reflect the non-static *in vivo* host response towards unfixed-tissue biomaterials. This information coupled with the impressive resistance unfixed bovine pericardium demonstrates towards cyclic dependent strain, makes unfixed bovine pericardium a potentially ideal biomaterial in heart valve engineering.

## Conclusion

The potential damage inflicted by the glutaraldehyde fixation process and the resultant limited longevity, inhibited recellularization capacity and inability to be remodeled, means superior alternatives to fixed-tissue bioprosthetic valves must be developed [5, 12, 41, 50]. The cyclic deformation induced changes in structure and function of glutaraldehyde-fixed tissue, demonstrated in the current study may ultimately be causative of structural valve deterioration in bioprosthetic heart valves, a possibility which warrants further investigation. Additionally, the findings of significant cycle dependent strain in porcine pericardium clearly highlight that uncycled uniaxial tensile testing alone is not sufficient to evaluate a tissue as a potential heart valve replacement. Candidate biomaterials should instead be assessed for cyclic dependent strain as a more accurate appraisal of their potential *in vivo* suitability. The current study also demonstrates that unfixed bovine pericardium not only resisted cyclic dependent strain up to 20 million cycles (equivalent of 6 months *in vivo*), but also maintained its structure, composition and mechanical function. Unfixed bovine pericardium may therefore represent a potentially ideal substrate for decellularization/antigen removal processes in the development of unfixed bioprosthetic heart valves. However, progress towards the use of unfixed-tissues as bioprosthetic heart valves, is likely to also require consideration of appropriate guidelines for how such materials are evaluated prior to *in vivo* implantation. Specifically, as this field continues to evolve, a more concrete understanding of the delicate balance between *in vivo* repair and remodeling of unfixed tissues compared to the rate of cyclic deformation induced biomaterial deterioration, will be required.

## Supporting information

S1 Fig Illustration of the length measurements on the samples to determine uniformity of the change in tissue dimensions due to fatigue loading in the heart valve tester. The uniformity of the sample loading was assessed by measuring the change in length of the sample at the nine points shown in the figure at baseline and after 10 million or 20 million cycles for glutaraldehyde fixed bovine pericardium. The variability in the change in length was then compared between these nine points to assess how fatigue varied across the sample and in turn how evenly loading was applied.
(DOCX)

S1 Table Mean and standard deviation (St. Dev.) calculated from the percentage change in dimensions of each GFBP sample at nine regions on the sample. **Changes in dimensions are calculated between baseline and 10 million cycles (10 MC), and baseline and 20 million cycles (20 MC).** The standard deviation is below 2% for all samples at both 10 million and 20 million loading cycles. However, the mean of all samples at 10 million cycles and 20 million cycles is also low and so it is not clear how much the standard deviation is affected by noise. Nevertheless, the variation in the strain is small and thus it may be assumed that loading that was applied by the heart valve tester was relatively uniform.
(DOCX)

S1 Supplementary Methods (DOCX)
