# Supplementary material for: Effect of cyclic deformation on xenogeneic heart valve biomaterials
Source: PLoS One. 2019 Jun 13;14(6):e0214656. doi: 10.1371/journal.pone.0214656 (PMC6563958; doi:10.1371/journal.pone.0214656)
Supplement: S1 Fig — The uniformity of the sample loading was assessed by measuring the change in length of the sample at the nine points shown in the figure at baseline and after 10 million or 20 million cycles for glutaraldehyde fixed bovine pericardium. The variability in the change in length was then compared between these nine points to assess how fatigue varied across the sample and in turn how evenly loading was applied. (DOCX) [file pone.0214656.s001.docx]

**Figures**

$$l_{1}^{1}$$

$$l_{2}^{1}$$

$$l_{3}^{1}$$

$$l_{1}^{2}$$

$$l_{2}^{2}$$

$$l_{3}^{2}$$

$$l_{1}^{3}$$

$$l_{2}^{3}$$

$$l_{3}^{3}$$

**S1 Fig. Illustration of the length measurements on the samples to determine uniformity of the change in tissue dimensions due to fatigue loading in the heart valve tester.** The uniformity of the sample loading was assessed by measuring the change in length of the sample at the nine points shown in the figure at baseline and after 10 million or 20 million cycles for glutaraldehyde fixed bovine pericardium. The variability in the change in length was then compared between these nine points to assess how fatigue varied across the sample and in turn how evenly loading was applied.
