# Supplementary material for: Effect of cyclic deformation on xenogeneic heart valve biomaterials
Source: PLoS One. 2019 Jun 13;14(6):e0214656. doi: 10.1371/journal.pone.0214656 (PMC6563958; doi:10.1371/journal.pone.0214656)
Supplement: S1 Table — Changes in dimensions are calculated between baseline and 10 million cycles (10 MC), and baseline and 20 million cycles (20 MC). The standard deviation is below 2% for all samples at both 10 million and 20 million loading cycles. However, the mean of all samples at 10 million cycles and 20 million cycles is also low and so it is not clear how much the standard deviation is affected by noise. Nevertheless, the variation in the strain is small and thus it may be assumed that loading that was applied by the heart valve tester was relatively uniform. (DOCX) [file pone.0214656.s002.docx]

**Tables**

**S1 Table. Mean and standard deviation (St. Dev.) calculated from the percentage change in dimensions of each GFBP sample at nine regions on the sample. Changes in dimensions are calculated between baseline and 10 million cycles (10 MC), and baseline and 20 million cycles (20 MC).** The standard deviation is below 2% for all samples at both 10 million and 20 million loading cycles. However, the mean of all samples at 10 million cycles and 20 million cycles is also low and so it is not clear how much the standard deviation is affected by noise. Nevertheless, the variation in the strain is small and thus it may be assumed that loading that was applied by the heart valve tester was relatively uniform.

| Sample | 1 | 2 | 3 | 4 | 5 | 6 |
| --- | --- | --- | --- | --- | --- | --- |
| Mean (10 MC) | 10.26% | 5.43% | 4.60% | 5.16% | 2.63% | 3.89% |
| St. Dev. (10 MC) | 1.94% | 1.01% | 1.07% | 1.73% | 0.46% | 0.80% |
| Mean (20 MC) | 2.83% | 2.59% | 2.74% | 2.87% | 3.28% | 2.49% |
| St. Dev. (20 MC) | 0.71% | 0.60% | 0.68% | 0.96% | 0.67% | 0.42% |
