# Supplementary material for: Effect of cyclic deformation on xenogeneic heart valve biomaterials
Source: PLoS One. 2019 Jun 13;14(6):e0214656. doi: 10.1371/journal.pone.0214656 (PMC6563958; doi:10.1371/journal.pone.0214656)
Supplement: S1 Supplementary Methods — (DOCX) [file pone.0214656.s003.docx]

**Supplementary material**

**Methods**

Unless stated otherwise, all chemicals were purchased from Sigma-Aldrich, St. Louis, MO, USA.

**Tissue harvest**

All experimental procedures and protocols were approved by the Mayo Clinic Institutional Animal Care and Use Committee (IACUC) and performed in accordance with the relevant guidelines and regulations from the Guide for the Care and Use of Laboratory Animals.[^34^](#_ENREF_34) Fresh bovine pericardium (BP) was harvested immediately postmortem from young adult cattle (Spear Products, Coopersburg, PA, USA), shipped on dry ice and stored at -80ºC upon arrival. Porcine pericardium (PP) was harvested immediately postmortem and stored in Dulbecco’s Modified Eagles Medium (DMEM) with 15% (v/v) dimethyl sulfoxide (DMSO) at -80°C. Both bovine and porcine tissue was defrosted and washed in 0.1% (w/v) anhydrous ethylenediaminetetraacetic acid (EDTA), 1% (v/v) antibiotic and antimycotic solution (AAS), phosphate-buffered saline (PBS) (pH 7.4) and 4% (v/v) Tris-HCl (pH 8.0). Tissue underwent dissection to remove connective tissue, and the pericardial sac was cut into 1.5x16 cm circumferential strips individually stored in DMEM with 15% (v/v) DMSO at -80°C. Glutaraldehyde-fixed bovine pericardium (GFBP) patches were purchased from Abbot, formerly St. Jude Medical, (St. Paul, MN) and stored at RT in provided storage solution as per manufacturer’s instructions.[^35^](#_ENREF_35)

**Assessment of variation in loading on the sample**

To verify if the loading was applied evenly we calculated the change in dimensions at nine regions along each GFBP sample compared to baseline (as a percentage) following 10 million or 20 million cycles of loading following the same procedure described in the Methods section for the whole sample (Supplementary Figure 1). The results of this are shown in supplementary table 1 where it can be seen that the standard deviation is below 2% for all samples at both 10 million and 20 million loading cycles. However, the mean of all samples at 10 million cycles and 20 million cycles is also low and so it is not clear how much the standard deviation is affected by noise. Nevertheless, the variation in the strain is small and thus it may be assumed that loading that was applied by the heart valve tester was relatively uniform.

**Quantitative biochemistry**

Sample lyophilization was performed on 6 mm discs using biopsy punches (Integra, Plainsboro, NJ) from each sample. Discs were weighed and lyophilized for 72 h at -56ºC and 0.27 Torr. Following lyophilization, discs were re-weighed and stored at -80°C. Tissue hydration was assessed by calculating the percent loss of sample mass following lyophilization (*n*=6 per group, per cycle).[^12^](#_ENREF_12)

Collagen content was assessed using the Collagen Hydroxyproline Assay (Chondrex Inc., Redmond, WA). Lyophilized samples underwent hydrochloric acid (HCL) digestion; incubation with 1 mL 5N HCl per 10 mg of lyophilized tissue, for 24 h at 120°C, and digested samples stored at -80°C. Briefly, 10 µL of distilled water (blank), type I collagen standards, and digested sample were combined with 100 µL of 1X Chloramine T Solution for 20 min at RT. Following incubation, 100 µL of Dimethylaminobenzaldehyde (DMAB) Solution was added and mixed at 300 rpm for 30 min at 60°C. Samples were read at 545 nm (*n*=6 per group, per cycle).[^12^](#_ENREF_12)

Elastin content was assessed using Fastin Elastin Assay (Bicolor Ltd., Carrickfergus, UK). Lyophilized samples underwent oxalic acid digestion; incubation with 1 mL 0.25M oxalic acid per 50 mg of lyophilized tissue for 60 min at 99°C and 300 rpm on a thermomixer. Samples were then centrifuged at 10,000 g for 10 min at RT and the supernatant collected. Oxalic acid digestion steps were repeated 3 times, with all supernatants pooled and stored at -80°C. In brief, 100 µL of oxalic acid (blank), elastin standard, and digested sample supernatant were combined with 100 µL elastin precipitating reagent and incubated for 15 min at RT, followed by centrifugation at 10,000 g for 10 min at RT. Supernatant was discarded and 1 mL dye reagent was added and mixed at 300 rpm for 90 min at RT. Samples were centrifuged at 10,000 g for 10 min at RT and the supernatant discarded. Dye dissociation reagent was added to each sample (250 µL) and incubated for 10 min at RT, the resulting supernatant was collected and dye absorbance read at 513 nm (*n*=6 per group, per cycle).[^12^](#_ENREF_12)
